# From gut colonization to recurrence: *E. coli* secretion systems at the host-microbiota interface

**DOI:** 10.1080/19490976.2026.2743921

**Published:** 2026-10-07

**Authors:** Marcin Równicki, Maciej Obrębski, Jakub P. Piwowarski

**Affiliations:** a Microbiota Lab, Department of Pharmaceutical Microbiology and Bioanalysis, Medical University of Warsaw, Warsaw, Poland

**Keywords:** *Escherichia coli*, gut microbiota, mucosal colonization, secretion systems, type III secretion system, type V secretion system, type VI secretion system, biofilm, recurrent infection, persister cells, interbacterial competition

## Abstract

*Escherichia coli* occupies a unique position between commensal colonization and infection. It is a common member of the intestinal microbiota, yet selected pathogenic or opportunistic lineages can persist in mucosal niches, interact with resident microbial communities, and in some cases seed recurrent extraintestinal diseases. The mechanisms enabling long-term colonization, survival under host and antibiotic pressure, and relapse are diverse, depending on pathotype, infection site, host environment, and microbial community structure. Here, we critically examine how T3SS, T5SS, and T6SS may contribute to persistence-associated biology in specific *E. coli* contexts. T3SS primarily mediates epithelial interaction in enteric pathotypes, T5SS drives adhesion, biofilm formation, mucosal colonization, and immune modulation, and T6SS functions as a microbiota-facing mechanism of interbacterial competition. We distinguish experimentally demonstrated roles from speculative links to chronicity and recurrence, framing these systems as context-dependent interaction modules rather than a unified network, while highlighting evidence gaps relative to established persistence determinants.

## I. Introduction

*Escherichia coli* is one of the most versatile bacterial species associated with humans and animals. It is a frequent member of the intestinal microbiota, but selected lineages have acquired virulence traits that allow them to cause disease in intestinal or extraintestinal sites. Pathogenic *E. coli* are commonly grouped into intestinal pathogenic *E. coli* (IPEC) and extraintestinal pathogenic *E. coli* (ExPEC).^1^ IPEC include enteropathogenic *E. coli* (EPEC), Shiga toxin-producing *E. coli* (STEC, including enterohemorrhagic *E. coli*, EHEC), enterotoxigenic *E. coli* (ETEC), enteroaggregative *E. coli* (EAEC), diffusely adherent *E. coli* (DAEC), and enteroinvasive *E. coli* (EIEC).^2^ ExPEC include uropathogenic *E. coli* (UPEC), neonatal meningitis-associated *E. coli* (NMEC), sepsis-associated *E. coli* (SePEC), and avian pathogenic *E. coli* (APEC).^1^ ^,^ ^3^ These groups differ substantially in ecological niche, infection route, tissue tropism, virulence repertoire, and clinical outcome.^3^ Therefore, mechanisms described in one pathotype or infection site should not be directly extrapolated to another without careful qualification. In this review, we focus primarily on EPEC, EHEC/STEC, EAEC, DAEC, and UPEC, for which secretion-system functions are directly relevant to the persistence-associated processes discussed below. The remaining pathotypes are included here to define the broader IPEC and ExPEC groups and are mentioned subsequently only where they provide mechanistically informative examples.

Long-lasting *E. coli*-associated conditions are biologically heterogeneous and differ across pathotypes and anatomical niches. For example, EAEC has been associated with persistent diarrhea,^4^ EHEC can show prolonged fecal shedding for weeks or, in some patients, months after symptomatic infection,^5^ DAEC has been linked to chronic mucosal inflammation,^6^ and UPEC can cause recurrent urinary tract infection or chronic bacterial prostatitis.^7^ These examples show that *E. coli* persistence can involve distinct processes, including prolonged colonization, chronic inflammation, and recurrent infection.^8^

In this review, we therefore use persistence-related terminology in a restricted sense (see Box 1). Persistent infection and recurrent infection are distinguished by whether bacterial presence continues with ongoing or intermittent pathology ^9^ or a new symptomatic episode occurs after apparent clinical resolution, respectively.^7^ Relapse is distinguished from reinfection according to whether recurrence originates from the same strain or reservoir or represents a new infection episode. Chronic infection involves ongoing bacterial presence or repeated reactivation,^10^ whereas chronic disease may persist after bacterial clearance because of host-driven inflammation, tissue remodeling, or immune dysregulation. Persister cells, biofilm-associated survival, and intracellular reservoirs are considered individually. These processes may overlap in some settings, but they are mechanistically distinct and should be interpreted separately unless direct evidence connects them.

Box 1. Glossary of persistence-related terms used in this review

**Table:** 

Term	Definition in this review.
**Colonization**	Presence and growth at a mucosal site without necessarily causing symptoms
**Persistent colonization**	Long-term bacterial presence at a site, often asymptomatic
**Persistent infection**	Continued bacterial presence with ongoing or intermittent pathology
**Recurrent infection**	New symptomatic episode after apparent clinical resolution
**Relapse**	Recurrence caused by the same strain/reservoir
**Reinfection**	New infection episode caused by a distinct strain
**Chronic infection**	Prolonged or fluctuating infection associated with ongoing bacterial presence or repeated reactivation
**Chronic disease**	Disease that may continue after bacterial clearance because of host-driven inflammation, tissue remodeling, or immune dysregulation.
**Persister cells**	Phenotypic antibiotic-tolerant subpopulation surviving bactericidal exposure without genetic resistance
**Biofilm-associated survival**	Protection caused by community structure, matrix, physiology, or gradients
**Intracellular reservoir**	Bacteria maintained within host cells, e.g. IBC/QIR in UPEC

Secretion systems are relevant to *E. coli* pathobiology because they mediate interactions with host cells, neighboring microbes, and the extracellular environment.^11^ They can deliver or export proteins that alter epithelial signaling, immune recognition, adhesion, aggregation, biofilm development, nutrient acquisition, or interbacterial competition.^12^ In this context, secretion systems should be viewed not only as acute virulence devices, but also as host-microbiota interaction modules that may support persistence and recurrence.

*E. coli* encodes or acquires multiple secretion and export pathways, including Type I, Type II, Type III, Type V, and Type VI secretion systems, as well as T4SS-like elements, outer membrane vesicles, and non-classical secretion processes.^13-15^ Type I and Type II secretion systems contribute to the export of several proteins relevant to bacterial physiology and virulence, and outer membrane vesicles can deliver toxins, enzymes, and immunomodulatory molecules. These mechanisms are important but are not the primary focus of this review. We focus on T3SS, T5SS, and T6SS because they map onto three major interaction axes at the host-microbiota interface: epithelial manipulation, mucosal adhesion or biofilm formation, and interbacterial competition.

T3SS is discussed primarily in the context of enteric pathotypes such as EPEC and EHEC, where it mediates intimate epithelial attachment, cytoskeletal remodeling, barrier disruption, and modulation of inflammatory or phagocytic responses. These activities can support colonization and mucosal niche formation, but direct evidence that T3SS drives chronic or recurrent *E. coli* infection remains context-dependent and limited. T5SS is discussed as a broad family of surface-associated and secreted proteins, including autotransporters, SPATEs, trimeric autotransporter adhesins, inverse autotransporters, and two-partner secretion systems. These proteins are strongly linked to adhesion, aggregation, mucin interaction, complement resistance, epithelial damage, and biofilm formation, all of which may contribute to mucosal persistence depending on pathotype and niche. T6SS is discussed mainly as a microbiota-facing system that mediates contact-dependent interbacterial competition, antibacterial effector delivery, immunity-dependent survival, and niche fitness in polymicrobial environments. Its contribution to persistence or recurrence in *E. coli* should be interpreted cautiously because it depends on strain background, effector repertoire, microbial community structure, and host environment.

The aim of this review is to provide a critical, context-separated synthesis of how secretion systems contribute to specific processes that may support long-term colonization, reservoir stability, antibiotic survival, or recurrence in defined *E. coli* pathotypes and infection models. We discuss evidence gaps that remain central to the field, including whether secretion systems are enriched in recurrent clinical isolates, whether they are expressed or active during chronic infection, whether they remain functional after antibiotic exposure, and how their contribution compares with established persistence determinants such as fimbriae, capsule, LPS remodeling, iron acquisition, toxins, stress-response pathways, c-di-GMP signaling, biofilm regulators, and metabolic adaptation.

## II. Secretion systems as host-microbiota interaction modules in *E. coli*

In the context of gut colonization and recurrent infection, secretion systems are relevant not simply because they deliver virulence factors, but because they mediate interactions with three major selective environments: host epithelial cells, immune defenses, and the resident microbiota. T3SS primarily shapes epithelial manipulation and host-cell interactions, T5SS contributes to adhesion, aggregation, mucosal persistence and biofilm formation, whereas T6SS supports interbacterial competition and niche fitness in polymicrobial environments.

(1) The Type III Secretion System (T3SS)

The Type III Secretion System (T3SS) is a multiprotein nanomachine used by certain pathogenic strains of *E. coli* to directly interface with host cells.^16^ This system enables the single-step transport of proteins across three barriers: the bacterial inner membrane, outer membrane, and host cell membrane (Figure 1).^17^ ^,^ ^18^ Proteins secreted by the T3SS, termed effector proteins, exhibit diverse functions aimed at subverting host cell processes to benefit bacterial survival and replication^19-22^ (Table 1). Effector proteins are recognized and secreted by the T3SS through an *N*-terminal signal sequence, which acts as a molecular tag to direct them to the secretion machinery.^23^ ^,^ ^24^ Prior to secretion, these effectors remain in their native, folded conformation. However, to traverse the narrow T3SS needle, they must undergo ATP-dependent unfolding—a prerequisite for their passage into the host cell.^25^ Many, but not all, T3SS effectors rely on dedicated bacterial chaperones during secretion to bind unfolded substrates, prevent premature folding and aggregation, and guide effectors to the injectisome.^26^ ^,^ ^27^ Once translocated into the host cytoplasm, effectors are intrinsically optimized to refold autonomously, owing to a unique balance of mechanical lability that enables their unfolding by the injectisome’s ATP-driven unfoldase and facilitates efficient spontaneous refolding within the host cell. Furthermore, pH-dependent folding switches in effectors promote conformational recovery after translocation without reliance on host chaperones, although some effectors may later hijack these chaperones to modulate host pathways.^28-30^

**Figure 1.**
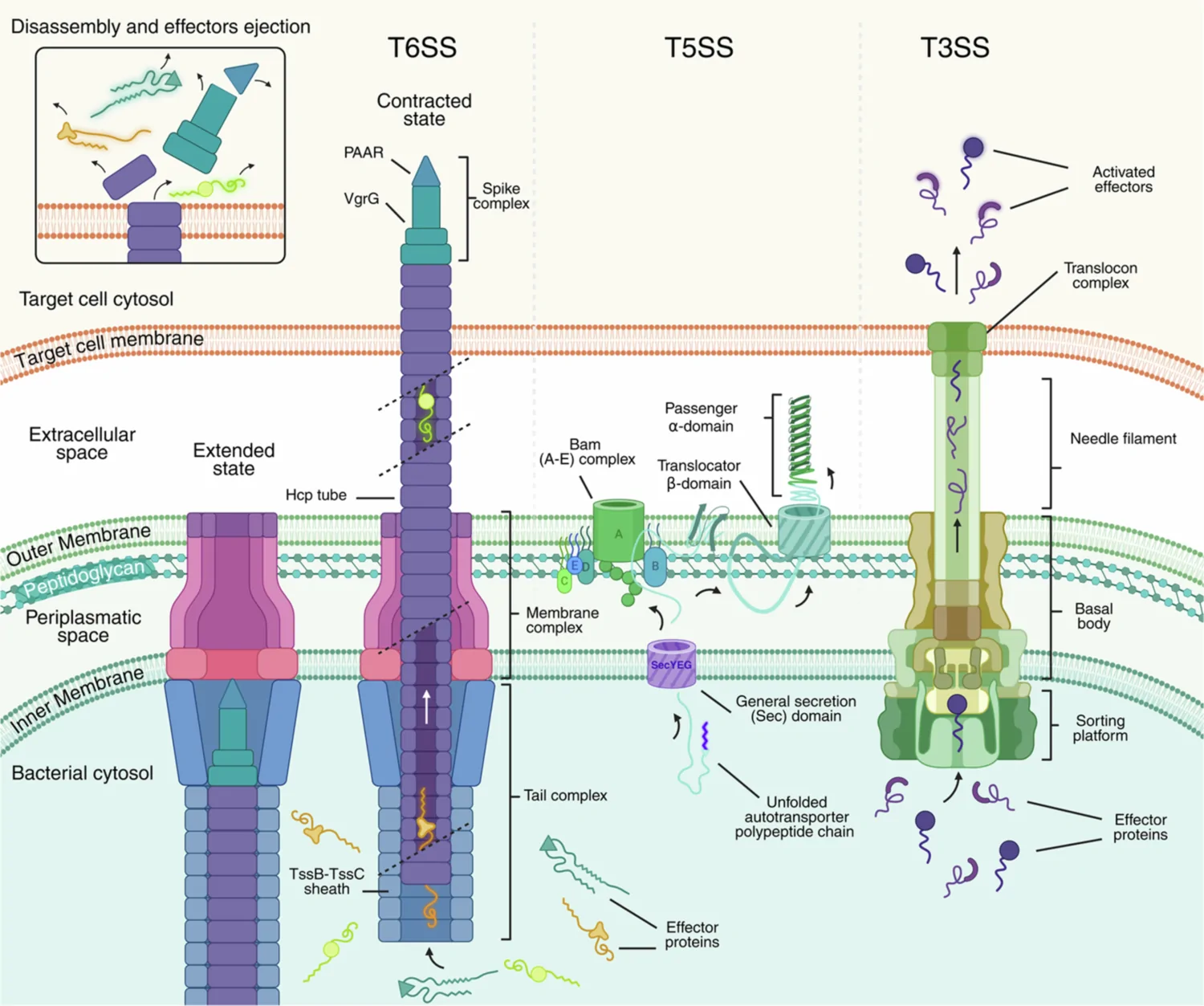
Schematic representation of Type III (T3SS), Type V (T5SS), and Type VI (T6SS) Secretion Systems. T3SS delivers effectors through an injectisome that spans the bacterial envelope and interfaces with the host membrane. T6SS is a contractile phage-tail-like apparatus that propels Hcp-VgrG-PAAR-associated effectors into neighboring cells or releases them extracellularly. In contrast, T5SS secretes proteins outside the cell through a two-step Sec-dependent mechanism. These proteins cross the inner membrane with the help of the Sec secretion pathways and then, after folding their translocator ß-domain in the outer membrane involving BAM complex, transport their own passenger *α*-domain to the extracellular space.

**Table 1.** Selected T3SS effectors from pathogenic *E. coli* and their reported functions with potential relevance to persistence-associated biology. Representative strains and experimental models are listed to indicate the biological context.

Pathotype and niche	Effector protein	Representative strain	Experimental model	Function	Persistence-relevant process	Evidence type	Reference
EPEC/EHEC—intestinal epithelium	EspF	EHEC O157:H7 EDL933	HT-29 intestinal epithelial cells;	Disrupts epithelial barrier, induces host-cell damage, and targets mitochondria	Barrier disruption may create a colonization-permissive mucosal interface; persistence link remains indirect	Acute virulence/indirect mechanistic evidence	[21]
	Tir	EPEC E2348/69 (O127:H6)	Epithelial-cell T3SS translocation and attachment assays	Acts as the intimin receptor and mediates actin pedestal formation	Stable epithelial attachment may support prolonged colonization, but not proven recurrence or chronic infection	Direct colonization evidence/indirect persistence relevance	[17]
	EspJ	EPEC E2348/69; EHEC O157:H7	Macrophage phagocytosis assays; opsonized sheep red blood cell assays	Inhibits Src signaling and phagocytosis by macrophages	Reduced phagocytic uptake may enhance survival during infection; persistence role remains inferred	Acute immune evasion/indirect persistence relevance	[31]
	EspG	EPEC E2348/69 (O127:H6)	Polarized MDCK epithelial monolayers; paracellular permeability assays	Modulates ARF GTPases, vesicle trafficking, and epithelial junctions	Barrier and trafficking disruption may support mucosal niche formation; no direct persistence phenotype shown	Acute epithelial manipulation/indirect evidence	[32]
	Map	EPEC E2348/69 (O127:H6)	HeLa and Caco-2 epithelial cells; mitochondrial targeting, Ca^2^⁺ signaling, and apoptosis assays	Activates Cdc42, induces filopodia, and may affect mitochondria	Cytoskeletal remodeling may facilitate epithelial colonization; persistence link is speculative	Acute host-cell manipulation/speculative persistence link	[33]
	EspH	EPEC E2348/69 (O127:H6)	Intestinal epithelial-cell junction and adhesion assays; murine colonization model	Inhibits RhoGEF-dependent Rho GTPase signaling and promotes desmosomal perturbation and barrier disruption	Altered epithelial junctions and cell–cell adhesion may affect mucosal colonization	Direct epithelial barrier and colonization evidence/indirect persistence relevance	[34]
	EspZ	EPEC E2348/69 (O127:H6)	Epithelial-cell infection, host-cell survival, and cytotoxicity assays	Modulates T3SS-dependent host interaction and limits excessive cytotoxicity	Limiting host-cell damage may help maintain an epithelial colonization niche; persistence benefit remains unproven	Virulence modulation/speculative persistence relevance	[35]
Atypical EPEC—intestinal epithelium	EspT	atypical EPEC E110019; JPN15 expressing EspT	HeLa, Swiss 3T3, and polarized Caco-2 cells; differential staining and gentamicin-protection invasion assays	Activates Rac1/Cdc42 signaling and induces membrane ruffling	EspT-dependent invasion and short-term intracellular survival have been demonstrated in vitro; no direct evidence links this phenotype to long-term reservoirs or recurrence	In vitro invasion phenotype/indirect evidence	[36]

T3SSs are structurally and functionally similar to a syringe and are often referred to as “injectisomes” and “needle and syringe”-like apparatuses because of their structure. At its core, the T3SS can be divided into three primary structural elements: a basal body, a needle component, and a translocon complex.^37^ ^,^ ^38^ The basal body, spanning both bacterial membranes, integrates cytoplasmic elements with a multi-ring architecture.^37^ That architecture bridges the inner and outer membranes, creating an anchor-like formation with a central rod. In most T3SSs, the basal body is comprised of over 15 distinct proteins.^39^ Extending outwards from the basal body, a hollow needle-like filament projects beyond the bacterial surface, passing through the secretin pore. This needle features a channel in the outer membrane, known as the secretin, and secretes effector proteins into the extracellular environment.^40^ Recent structural studies provide the first direct proof that translocation occurs via passage through the needle's hollow core by imaging a “trapped” effector protein within the needle using cryo-EM and single-particle techniques.^18^

At the needle tip resides the translocon complex, a critical component that detects host cells, mediates interaction, and regulates effector release.^41^ When bacteria contact a host cell, translocons assemble and form a pore that delivers and allows effector proteins to enter and act on the host cell.^42^ ^,^ ^43^ Once assembled, the T3SS functions as a molecular syringe, injecting effector proteins into the host cell cytoplasm.^44^

The translocon is crucial for this delivery step, but it is not involved in the effectors' initial release from the bacteria.^45^ Some studies, however, challenge this view, proposing a revised two-step model instead. In this model, the effectors and the translocon components are released prematurely and stored near the bacterial surface, potentially within lipid vesicles, before host contact.^42^ ^,^ ^43^

Contact of bacteria with the host cell, possibly mediated by the needle, triggers pore formation by these pre-secreted components, allowing effectors to enter the host.^42^ ^,^ ^43^ However, further research is necessary to determine the exact mechanism driving this process.

Interestingly, the T3SS injectisome shares a conserved transmembrane export apparatus with the flagellum, including homologous basal body rings and a protein secretion mechanism driven by ATP hydrolysis and proton motive force.^46^ Phylogenetic evidence indicates that injectisomes evolved from flagellar systems through gene loss and horizontal transfer, adapting the motility machinery for effector protein translocation into host cells.^47^ Moreover, there is evident regulatory interplay between these two components. Shulman et al. demonstrated that functional T3SS influences flagellar motility through transcriptional regulation of flagellar genes and secretion of flagellar proteins.^48^ Notably, wild-type strains maintain motility through T3SS-mediated flagellar protein secretion, while T3SS mutants exhibit reduced transcription of flagellar genes and impaired motility. Degenerate T3SS loci, although no longer functioning as canonical secretion systems, may contribute to pathogenesis by altering bacterial surface properties, adhesion, or host interactions.^48^ Within the gut ecosystem, T3SS activity is therefore best understood as an epithelial interaction module. Its major contribution to persistence may not be chronicity per se, but the creation of a permissive mucosal interface through intimate attachment, barrier disruption, immune modulation, and altered epithelial signaling.

(2) The Type V Secretion System (T5SS)

Type V secretion systems (T5SS) are among the most prevalent mechanisms used by Gram-negative bacteria to display or secrete surface-associated proteins, including many *E. coli* colonization and virulence factors.^14^ ^,^ ^49^ In *E. coli*, T5SS proteins generally follow a Sec-Bam-dependent pathway: precursor polypeptides cross the inner membrane through the Sec pathway, are stabilized in the periplasm, and are assembled in the outer membrane as *β*-barrel translocator domains that deliver passenger domains to the bacterial surface or extracellular space (Figure 1).^50^ ^,^ ^51^

T5SS proteins are structurally diverse, comprising several subfamilies from Type Va to Type Ve. Because detailed T5SS classification and transport mechanisms have been reviewed extensively elsewhere, including in T5SS-focused reviews by Fan et al.^14^ and Meuskens et al.^52^, we discuss architecture here only where it is directly relevant to adhesion, immune modulation, biofilm formation, mucosal colonization, or interbacterial competition. Accordingly, we focus on the T5SS subfamilies most relevant to *E. coli* persistence-associated biology: classical autotransporters (Va), two-partner secretion systems (Vb), trimeric autotransporters (Vc), and inverse autotransporters (Ve).^49^ ^,^ ^53^

Within the Va group, self-associating adhesins such as Ag43, AIDA-I, TibA, and Upa family proteins promote autoaggregation, epithelial or extracellular-matrix adhesion, and biofilm formation in pathotypes including DAEC, EAEC, STEC, and UPEC (Table 2).^54-56^ Serine protease autotransporters of Enterobacteriaceae (SPATEs), including EspP, Pic/PicU, Sat, Vat, and Hbp, act on host substrates such as mucins, complement components, coagulation factors, and leukocyte glycoproteins, depending on the protein (Table 2).^57-59^ Through these activities, SPATEs can modulate mucus barriers, serum killing, epithelial integrity, and inflammatory responses. These functions may support mucosal niche formation or prolonged colonization in specific contexts, but their contribution to long-term carriage or recurrence remains pathotype- and model-dependent and has not yet been directly demonstrated in most *E. coli* systems.

**Table 2.** Representative T5SS proteins or protein groups in *E. coli* and their reported functions with potential relevance to persistence-associated biology. Representative strains and experimental models are listed to indicate the biological context of the cited evidence. An asterisk indicates proteins reported in both commensal and pathogenic *E. coli*.

T5SS subtype	Representative protein(s)	Pathotype and niche	Representative strain	Experimental model	Function	Persistence-relevant process	Evidence type	Reference
Va—self-associating autotransporters (SAATs)	Ag43*	UPEC—urinary tract	UPEC CFT073	In vitro autoaggregation and biofilm assays; murine urinary tract colonization and long-term persistence models	Autoaggregation and biofilm formation	Ag43-dependent aggregation and biofilm formation can support urinary tract colonization; long-term persistence has been demonstrated in a UPEC model, whereas a direct role in recurrence remains unproven	Direct biofilm and in vivo persistence evidence/recurrence relevance unproven	[54,60]
Va—self-associating autotransporters (SAATs)	AIDA-I, TibA	Diarrhoeagenic *E. coli*/ETEC—intestinal niche	AIDA-I-positive strain 2787 (O126:H27); TibA-positive diarrhoeagenic *E. coli*	In vitro bacterial aggregation, epithelial adhesion, and biofilm assays	Self-recognition, autoaggregation, epithelial adhesion, and biofilm formation	May support intestinal colonization and community-associated protection; direct evidence for long-term carriage or recurrence is lacking	Direct adhesion and biofilm evidence/indirect persistence relevance	[61–65]
Va—Upa-family autotransporter adhesins	UpaB*, UpaC, UpaH	UPEC—urinary tract	UPEC CFT073	ECM- and epithelial-adhesion assays; in vitro biofilm assays; murine bladder colonization models	Adhesion to host surfaces and ECM components; biofilm formation	Supports bladder colonization and surface-associated growth in UPEC models; direct links to recurrent infection remain unproven	Direct adhesion, biofilm, and colonization evidence/indirect recurrence relevance	[56,66]
Va—SPATEs: complement and host-substrate modulation	EspP	STEC/EHEC—intestinal niche	EHEC O157:H7	Biochemical substrate-cleavage assays; complement assays; bacterial aggregation and adherence models	Cleavage of host substrates and complement components; modulation of adherence-associated phenotypes	Complement modulation and altered adherence may favor survival or colonization in enteric infection; direct evidence for persistent carriage or recurrence is lacking	Direct enzymatic and immune-modulation evidence/indirect persistence relevance	[58,67–70]
Va—SPATEs: mucus and complement modulation	Pic/PicU	EAEC—intestinal mucus; UPEC—urinary or extraintestinal niche	EAEC 042 and other Pic-positive EAEC, PicU-positive UPEC strains	Mucin degradation and goblet-like epithelial-cell assays; complement and serum-resistance assays; leukocyte-glycoprotein cleavage assays	Mucin degradation and secretion modulation; complement cleavage; leukocyte-glycoprotein processing	May facilitate mucus-layer access and immune modulation in specific pathotype contexts; direct long-term persistence or recurrence evidence remains limited	Direct mucinase and immune-modulation evidence/indirect persistence relevance	[57,59,71,72]
Va—SPATEs: epithelial injury/host-substrate cleavage	EspC	EPEC—intestinal epithelium	EPEC E2348/69	Intestinal epithelial-cell and host-substrate proteolysis assays	Proteolytic activity and epithelial injury	Epithelial damage may modify the mucosal environment during infection; a direct persistence phenotype has not been demonstrated	Acute virulence/mechanistic evidence/speculative persistence relevance	[73]
Va—SPATEs: epithelial injury	Sat	DAEC and UPEC—intestinal or urinary epithelium	Afa/Dr fimbriae expressing DAEC Sat-positive strains; UPEC CFT073; EPEC E2348/69	Polarized epithelial monolayers; bladder and kidney epithelial-cell models	Tight-junction disruption, vacuolation, and epithelial damage	May alter epithelial integrity and promote survival or dissemination in experimental models; direct roles in intracellular reservoirs or recurrence remain unproven	Direct epithelial injury evidence/indirect persistence relevance	[74–76]
Va—SPATEs: epithelial injury	Vat	APEC and UPEC/ExPEC—extraintestinal or urinary epithelium	Vat-positive APEC and UPEC/ExPEC	Bladder epithelial-cell infection and cytoskeletal-disruption assays	Vacuolation, cytoskeletal remodeling, and epithelial injury	May create host-cell conditions favorable to bacterial survival or dissemination; direct QIR, reservoir, or recurrence evidence is lacking	Acute epithelial manipulation/indirect persistence relevance	[77,78]
Va—SPATEs: host-substrate cleavage and nutrient acquisition	Hbp/Tsh	ExPEC and APEC—extraintestinal or inflamed niches	Hbp/Tsh-positive ExPEC and APEC strains	Biochemical proteolysis, hemoglobin/heme acquisition, and leukocyte-glycoprotein cleavage assays	Host-protein cleavage and heme-associated iron acquisition	May support survival in iron-limited or inflamed environments; a direct contribution to persistent or recurrent infection has not been demonstrated	Direct biochemical and nutrient-acquisition evidence/indirect persistence relevance	[79–82]
Va—SPATEs: epithelial interaction	Sha, TagB, TagC	ExPEC—urinary epithelium	ExPEC isolates expressing Sha, TagB, or TagC	Human bladder epithelial-cell internalization and cytoskeletal-disruption assays	Internalization by epithelial cells and actin cytoskeletal disruption	Host-cell interaction may influence epithelial colonization or survival; long-term reservoir and recurrence roles remain unproven	In vitro epithelial interaction evidence/speculative persistence relevance	[83]
Vb—two-partner secretion	CdiA/B*	Commensal *E. coli* and UPEC—high-density bacterial communities	Commensal gut isolate EC93; UPEC 536	High-density clonal coculture, contact-dependent inhibition, and antibiotic-persister assays	Contact-dependent inhibition and toxin delivery to neighboring or sibling bacteria	CDI directly increases antibiotic-tolerant persister-cell levels under defined high-density conditions; roles in intracellular reservoirs, treatment failure, or recurrence remain unproven	Direct in vitro CDI–persister evidence/broader clinical relevance indirect	[84,85]
Vc—trimeric autotransporter adhesins	EhaG, UpaG	EHEC and UPEC—epithelial or ECM-associated surfaces	EHEC O157:H7; UPEC CFT073	ECM-binding, autoaggregation, and in vitro biofilm assays; UPEC colonization-associated models	Multivalent adhesion to ECM, aggregation, and biofilm formation	May support stable surface colonization and biofilm-associated growth; persistence beyond colonization remains indirect	Direct adhesion and biofilm evidence/indirect persistence relevance	[86,87]
Ve—inverse autotransporters	Intimin	EPEC/EHEC—intestinal epithelium	EPEC E2348/69 (O127:H6); EHEC O157:H7	Epithelial-cell attachment and actin-pedestal assays; attaching-and-effacing lesion models	Intimate attachment through binding to T3SS-delivered Tir and actin-pedestal formation	Strongly supports epithelial colonization; chronicity and recurrence have not been directly demonstrated	Direct colonization and virulence evidence/indirect persistence relevance	[88–90]
Ve—inverse autotransporters	YeeJ*	Commensal and pathogenic *E. coli*—surface-associated niches	*E. coli* K-12 MG1655	In vitro biofilm, surface-adhesion, and peptidoglycan-binding assays	Surface adhesion and biofilm formation	May promote surface-associated niche stability in specific strain backgrounds; direct long-term colonization or recurrence evidence is lacking	Direct in vitro adhesion and biofilm evidence/indirect persistence relevance	[49,53]

A distinct functional axis of T5SS is interbacterial competition. Among non-classical Type V systems, two-partner secretion systems such as CdiA/B mediate contact-dependent inhibition of neighboring bacteria and have been linked to increased levels of antibiotic-tolerant persister cells in UPEC under defined experimental conditions, suggesting a possible density-dependent survival strategy in competitive niches (Table 2).^84^ ^,^ ^85^ T5SS proteins can also reinforce physical retention at host surfaces. Trimeric autotransporters (Vc), exemplified by EhaG and related adhesins, form multimeric surface structures specialized for strong adhesion to host tissues and extracellular matrix, and can contribute to biofilm formation in EHEC and UPEC.^86^

Likewise, inverse autotransporters (Ve) contribute to host-surface attachment. These include intimin, a key adhesin of EPEC and EHEC that mediates intimate attachment to intestinal epithelium through binding to the T3SS-delivered Tir receptor,^88^ ^,^ ^89^ and YeeJ, which supports mucosal adhesion and biofilm formation (Table 2).^53^Unlike T3SS and T6SS, many T5SS proteins are shared between commensal and pathogenic *E. coli*, and their biological impact depends strongly on regulatory and ecological context. This is particularly relevant for ExPEC lineages, which can reside in the gut as commensals but cause disease after translocation to extraintestinal sites. The examples above illustrate several distinct functional routes by which T5SS proteins may support niche establishment: surface-associated adhesins promote aggregation, epithelial or extracellular-matrix attachment, and biofilm-associated retention; SPATEs modify host-facing barriers and immune components; and CdiA/B primarily targets neighboring bacteria, linking T5SS activity to interbacterial competition. Regulatory plasticity further shapes these functions, as illustrated by phase-variable Ag43, context-dependent Upa proteins, and CdiA/B-mediated competition.^56^ ^,^ ^91^ ^,^ ^92^ Thus, T5SS contributions to persistence-associated biology arise from mechanistically distinct activities rather than from a single conserved persistence function.

Taken together, T5SS proteins act at the boundary between commensal fitness and virulence. Their roles in adhesion, aggregation, mucin interaction, complement modulation, interbacterial competition, and biofilm formation provide plausible routes toward mucosal persistence or protected-niche survival. However, these activities should not be interpreted as direct evidence for chronic or recurrent infection unless tested in appropriate long-term colonization, antibiotic-survival, reservoir, or relapse models.

(3) The Type VI Secretion System (T6SS)

The Type VI Secretion System (T6SS), identified after the classical Type I-V secretion systems, is a contractile phage-tail-like apparatus widely distributed among Gram-negative bacteria.^93^ These gene clusters, averaging 20 kb in size, often exist in multiple chromosomal copies.^94^ Initially hypothesized to act as a virulence mechanism targeting eukaryotic host cells—a role supported by its involvement in pathogenesis—only a subset of T6SSs have been linked to host infection.^95^ Instead, evidence highlights its critical role in bacterial competition: the T6SS delivers antibacterial effectors directly into neighboring cells during cell-to-cell contact. Target-cell survival depends largely on whether the recipient encodes cognate immunity proteins or other protective mechanisms. In *E. coli,* several T6SS effector proteins have been identified.^96-98^

In addition to its antibacterial activity, the T6SS can target eukaryotic cells, including fungal cells, expanding its role in inter-kingdom interactions (Table 3).^15^ ^,^ ^95^ In some bacteria, T6SS-associated effectors have been implicated in nutrient acquisition, including acquisition of metal ions such as iron and manganese. Whether this mechanism broadly contributes to *E. coli* persistence remains less well established.^99^ This functional versatility highlights the adaptability of the T6SS and its importance in bacterial survival and fitness.

**Table 3.** Representative T6SS effectors, T6SS-associated proteins, or system-level T6SS phenotypes in *E. coli* and their reported functions with potential relevance to persistence-associated biology. Representative strains and experimental models are listed to indicate the biological context of the cited evidence.

Pathotype and niche	Effector/protein	Representative strain	Experimental model	Function	Persistence-relevant process	Evidence type	Reference
EAEC—intestinal/polymicrobial niche	AaiC (Hcp-like T6SS-associated protein)	EAEC 042	Comparative proteomic and microarray analysis of the AggR regulon; secreted-protein profiling	Secreted Hcp-like T6SS-associated component; precise toxic effector activity remains unknown	Demonstrates regulated T6SS-associated secretion, but does not establish a persistence phenotype	Secretion and regulatory evidence	[100]
EAEC—bacterial competition/polymicrobial niche	Tle1	EAEC 17-2	T6SS-dependent bacterial competition assays with *E. coli* prey; biochemical phospholipase assays; VgrG-Tle1 interaction studies	Phospholipase A1/A2 activity and VgrG1-dependent delivery to prey cells, causing membrane disruption	May support interbacterial competition and niche fitness in polymicrobial communities; long-term carriage or recurrence has not been demonstrated	Direct antibacterial effector evidence/ecological persistence relevance inferred	[101]
*E. coli*—bacterial competition	Rhs-CT3, Rhs-Nb-CT5	ETEC PE027	T6SS-dependent interbacterial antagonism; effector–immunity and delivery assays	Rhs-CT3 carries a REase-3 nuclease domain and mediates interbacterial antagonism; Rhs-Nb-CT5 mediates T6SS-dependent antibacterial activity via the Hcp2B–VgrG2 module, whereas its proposed RNase activity remains unverified	Antibacterial toxicity may promote competitive fitness and niche occupation; direct persistence or recurrence evidence is lacking	Direct antibacterial effector evidence/persistence relevance indirect	[102]
STEC/ETEC—bacterial competition	Hcp-ET1, Hcp-ET2, Hcp-ET3/ET4	STEC004 (Hcp-ET1); ETEC PE321 (Hcp-ET2); ETEC PE086 (Hcp-ET3/ET4-associated module)	Contact-dependent bacterial competition; effector–immunity assays; purified-protein nuclease and interaction assays	Hcp-ET1 exhibits HNH-DNase-associated toxicity; Hcp-ET2 has Tle1-family phospholipase activity; Hcp-ET3/ET4 mediate Pyocin S3- and Colicin-DNase-associated antibacterial effects	Direct killing of bacterial competitors may promote niche occupation under competitive conditions; relevance to long-term colonization remains untested	Direct antibacterial effector evidence/ecological persistence relevance inferred	[98]
EHEC—macrophage oxidative-stress environment	KatN	EHEC O157:H7; EDL933	RAW264.7 macrophages; primary murine peritoneal macrophages; oxidative-stress survival assays; murine oral infection model	T6SS-dependent secretion of Mn-containing catalase reduces intracellular ROS and enhances survival in macrophages	Supports intramacrophage survival under oxidative stress in a specific EHEC model; no direct evidence for chronic infection or recurrence	Direct host-cell survival evidence/persistence link context-dependent	[95]
Porcine ExPEC—extraintestinal host-cell niches	Hcp1, Hcp3	Porcine ExPEC PCN033	PK-15 epithelial cells; porcine alveolar macrophages; murine organ-colonization and bacteraemia model	Predominantly contribute to bacterial–eukaryotic cell interactions and host-cell-associated phenotypes	May support host-cell interaction and extraintestinal colonization in the PCN033 background; a direct recurrence role has not been demonstrated	Direct host-interaction and colonization evidence	[103]
Porcine ExPEC—bacterial competition	Hcp2	Porcine ExPEC PCN033	In vitro bacterial competition assays; murine organ-colonization model	Predominantly contributes to interbacterial competition; Hcp-family proteins also influence PCN033 colonization phenotypes	May enhance competitive fitness in the PCN033 background; broader relevance to gut reservoirs or persistent infection remains uncertain	Direct competition and strain-specific colonization evidence	[103]
Porcine ExPEC—bacterial and host interaction contexts	VgrG1	Porcine ExPEC PCN033	In vitro bacterial competition and host-cell interaction assays; murine infection model	Contributes to antibacterial interactions and virulence-associated host-cell phenotypes	May support strain-specific competitive fitness and virulence; a direct role in chronic or recurrent infection remains unproven	Direct competition and virulence evidence/persistence link indirect	[104]
Commensal *E. coli*—murine gut/polymicrobial niche	T6SS-1 and T6SS-2 (system-level phenotype)	Commensal *E. coli* Mt1B1	In vitro interbacterial competition; murine gut colonization and competition with *Citrobacter rodentium*	T6SS-dependent countercompetition against an enteric pathogen in the gut	Directly supports microbiota-facing niche competition and gut niche occupancy; this phenotype should not be extrapolated to clinical recurrence	Direct in vivo gut competition and niche-occupancy evidence	[105]

The T6SS apparatus is a double-membrane-spanning structure that delivers bacterial cytoplasmic substrates directly into a target cell or to the extracellular space (Figure 1).^106^ This transport mechanism is a one-step process where substrates are secreted across both bacterial membranes simultaneously. Its architecture comprises three primary structural modules: a membrane complex, a baseplate complex, and a tail complex.^107^

The membrane complex acts as an anchor, facilitating the integration of the T6SS into the bacterial cell envelope. This complex is specific to the T6SS and is minimally composed of three proteins: TssJ (an outer membrane lipoprotein), TssL (an inner membrane protein), and TssM (an inner membrane connector). Together, these proteins form a channel through which the tail complex can pass. The membrane complex is also crucial for maintaining the integrity of the bacterial cell during the secretion process. TssM is a key component that connects the outer membrane protein TssJ and the inner membrane protein TssL.^107^ The next component, the baseplate, is composed of five cytoplasmic proteins—TssE, TssF, TssG, TssA, and TssK—and connects the membrane complex (TssJ–TssL–TssM) to the tail components. This assembly platform anchors the tail and initiates its polymerization. The sixth protein, VgrG, forms a needle-like spike in the center of the baseplate complex and is required for proper assembly of the T6SS tail tube. VgrG, located at the tip of the tail, helps to penetrate the target cell.^96^ ^,^ ^108^

Extending from the baseplate is the contractile tail sheath, a tubular structure formed by TssB and TssC proteins.^109^ This sheath spans from the bacterial inner membrane into the cytoplasm, encapsulating an inner tube composed of hemolysin co-regulated protein (Hcp). The Hcp tube, assembled as stacked hexameric rings, serves as an effector carrier or scaffold for transporting effector proteins—either folded or partially unfolded—into target cells during sheath contraction.^102^ ^,^ ^103^ Together, the sheath and inner tube form a dynamic, phage-like apparatus optimized for precise effector delivery.^110^

The assembly and function of the T6SS rely on several other proteins. These include ClpV ATPase (also known as TssH), which facilitates the depolymerization of TssB/TssC complexes within the cytoplasm; TssE, which initiates sheath polymerization; and PAAR, which sharpens the tip of the VgrG spike and may connect VgrG to effector proteins.^97^ ^,^ ^111^ The structural architecture of the T6SS apparatus has been described in detail by structural and imaging studies^106^ ^,^ ^108^ ^,^ ^112^ and reviewed for pathogenic *E. coli*.^113^

The T6SS operates through a series of steps. First, the components assemble at the bacterial membrane, with the sheath forming around the inner tube and the baseplate attaching to the sheath. Upon firing, the extended sheath rapidly contracts, propelling the Hcp tube and VgrG spike out of the bacterial cell and into the target cell.^112^ After contraction, the ATPase ClpV disassembles the contracted TssB/TssC sheath to recycle its components for another firing cycle.^97^ ^,^ ^111^

The VgrG spike helps penetrate the target cell membrane, and the effector proteins are delivered through the inner tube. Some VgrG proteins have extensions that act as effector domains, allowing the T6SS to deliver a variety of different proteins. This versatility makes the T6SS a powerful tool used by bacteria for both offense and defense. It can inject toxins into competing bacteria or deliver proteins that help the bacteria survive in harsh environments.^105^ ^,^ ^112^ ^,^ ^114^

The T6SS displays structural and mechanistic similarities to bacteriophage tail structures, with VgrG proteins mimicking the gp5-gp27 puncturing complex of phage T4 and Hcp hexamers forming tubes analogous to viral tail tubes.^106^ Cryo-electron tomography reveals that T6SS sheaths adopt extended and contracted states nearly identical to phage tails, with contraction propelling Hcp-VgrG complexes into adjacent cells.^112^ While phages use this machinery for DNA delivery, bacteria repurposed it for interbacterial competition and host manipulation, retaining core structural motifs but adapting regulatory elements for environmental responsiveness.^93^ In the gut, the relevance of T6SS is therefore primarily ecological. By mediating contact-dependent interactions with neighboring microbes, T6SS may influence niche occupation, competition within polymicrobial communities, and the stability of *E. coli* reservoirs. Changes in the relative abundance and spatial occupancy of competing bacterial populations may in turn modify the microbial environment encountered at the mucosal surface, providing an indirect route through which T6SS-mediated competition can affect the host-microbiota interface. Its contribution to host virulence appears more strain-dependent and should therefore be interpreted more cautiously than its role in interbacterial competition.

This ecological role is illustrated *in vivo* by the interaction between the murine pathogen *Citrobacter rodentium* and commensal *E. coli* Mt1B1. A study by Serapio-Palacios et al. showed that *C. rodentium* uses T6SS-1 to target commensal *E. coli* during intestinal colonization, whereas *E. coli* Mt1B1 employs two T6SSs of its own to counter this competition.^105^ This interaction demonstrates how T6SS activity can shape gut niche occupancy by determining which bacterial populations retain access to colonized intestinal niches. It also provides an intriguing example of how *E. coli* can use T6SS not only for offensive competition but also for defense against competing bacteria, highlighting reciprocal competitive dynamics within the gut microbiota.

Although T3SS, T5SS, and T6SS differ in architecture, substrates, and mechanisms of transport, their activities can affect related infection-associated processes, including epithelial interaction, immune modulation, biofilm formation, and interbacterial competition. More specifically, T3SS primarily acts at the epithelial interface through host-cell manipulation, T5SS supports surface retention and host-facing interactions through adhesion, aggregation, biofilm formation, and secreted or surface-associated proteins, whereas T6SS primarily shapes the microbial neighborhood through contact-dependent competition and niche occupancy. Together, these distinct activities provide complementary routes by which *E. coli* can establish and maintain colonized niches in a pathotype- and context-dependent manner. However, overlap at the phenotypic level should not be interpreted as evidence that these systems are co-regulated or function as a coordinated persistence network. The following sections therefore discuss these functions by biological context and distinguish experimentally demonstrated mechanisms from indirect or speculative links to persistence-associated biology.

## III. Secretion-system functions at the gut-host interface

*E. coli* can establish long-term colonization or persistent infection in specific pathotypes, niches, and host contexts. Secretion systems may contribute to several processes associated with long-term survival within the host. In contexts where persistence-associated biology is relevant, three broad processes are particularly relevant: (i) modulation or evasion of host immune defenses, (ii) mucosal niche establishment and maintenance, and (iii) survival of antibiotic exposure. Below we explore the complex interplay between *E. coli* T3SS, T5SS, and T6SS and these steps, focusing on how these systems may contribute to chronic infection, recurrence or persister cell-associated survival.

(1) Modulation of the host immune defenses

The host immune system is a main barrier against bacterial pathogens, hindering their ability to establish infection and spread. *E. coli* has evolved mechanisms to modulate innate immune responses, including strategies to evade phagocytosis and autophagy, which ultimately allows *E. coli* to survive and proliferate within the host.

*T3SS*

Phagocytosis serves as a key defense mechanism of the innate immune system, enabling the engulfment and destruction of invasive pathogens. *E. coli*, mainly EHEC and EPEC, employ a T3SS to subvert this process, evading both cis-phagocytosis (direct uptake of the bacterium itself) and trans-phagocytosis (phagocytosis of unrelated IgG-opsonized particles)^115^ through modulation of Rho family of small GTPases and other actin regulators. These effectors primarily illustrate how T3SS can modulate phagocytic uptake and epithelial responses during intestinal infection. The T3SS-dependent effectors—EspF, EspB, EspJ, and EspH—have been characterized as key inhibitors of phagocytosis.^19^ ^,^ ^20^ ^,^ ^115^ EspF inhibits PI3K (phosphatidyl inositol 3-kinase) activation,^115^ and EspJ inhibit the Src signaling, both of which are required for phagocytosis.^20^ In addition, studies have shown that EspF inhibits phagocytosis by macrophages ^116^ by disrupting mitochondrial activity, and nucleolar function (Figure 2).^21^ EspB affects myosin function to inhibit pseudopod extension and phagosome closure (Figure 2),^117^ EspH disrupts the host-cell actin cytoskeleton by suppressing Rho GTPases,^34^ crucial regulators of actin polymerization. This disruption impairs the ability of phagocytes to engulf bacteria by targeting Rho guanine nucleotide exchange factors (GEFs), which activate Rho GTPases.^19^ By inhibiting RhoGEFs, EspH prevents the activation of Rho GTPases and subsequent actin polymerization, preventing bacterial internalization.^19^ Of note, EspT expressed by atypical invasive EPEC, exhibits GEF activity that induces the formation of actin-based plasma membrane ruffles, thereby facilitating the bacterium’s invasion of nonphagocytic cells.^36^T3SS effectors can also modulate inflammatory signaling pathways, including nuclear factor-κB (NF-κB) and mitogen-activated protein kinase (MAPK); these mechanisms are not discussed in detail here because they have been extensively reviewed elsewhere.^118^ ^,^ ^119^

**Figure 2.**
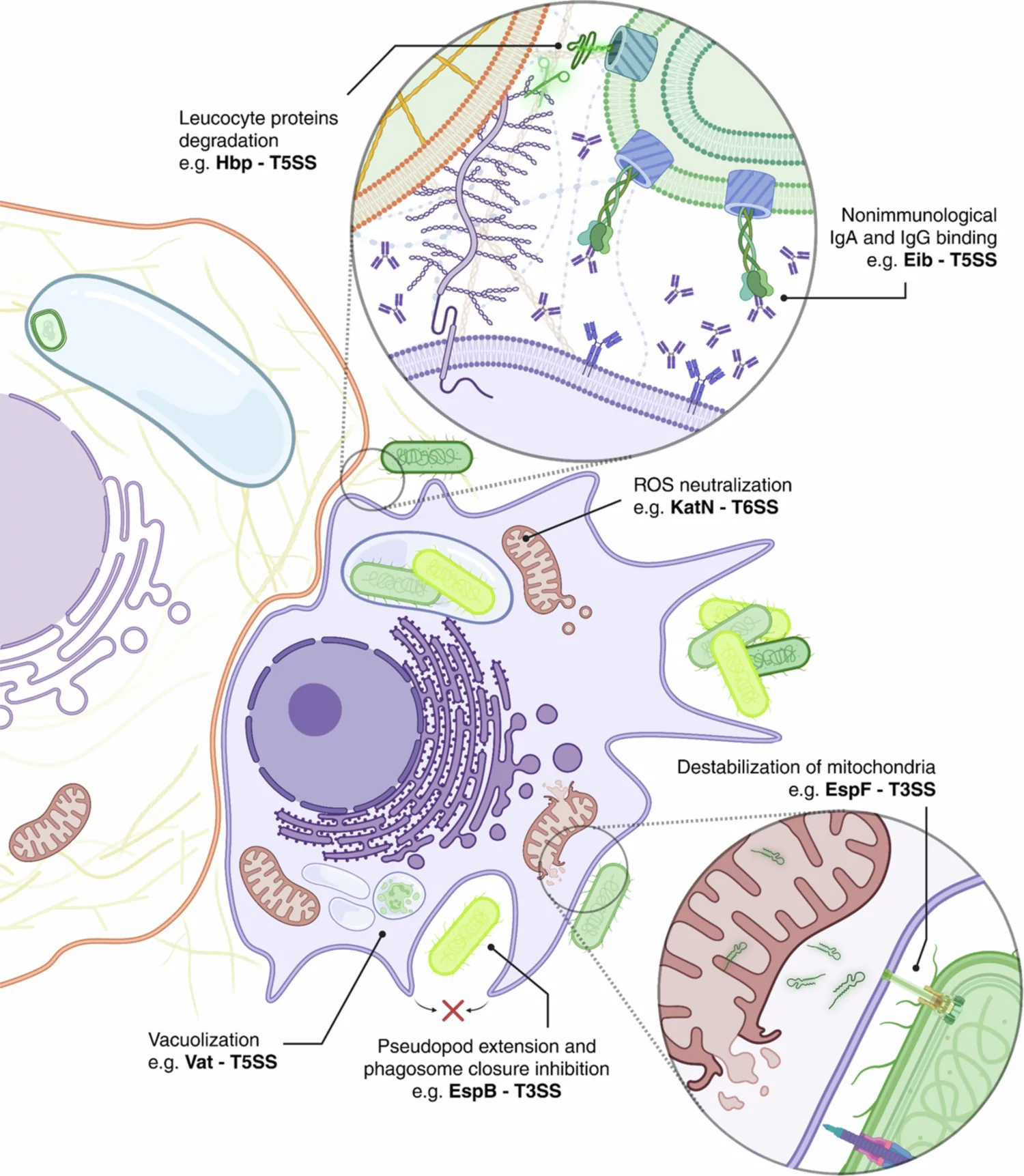
Mechanisms by which *E. coli* secretion-system-associated effectors modulate innate immune responses. The figure illustrates selected T3SS, T5SS, and T6SS-associated activities involved in epithelial damage, complement or antibody interactions, ROS neutralization, and phagocytosis-related processes.

*T5SS*

Two well-described autotransporters (ATs) from the Va subtype of the SPATE group: extracellular serine protease *P* (EspP) and the protein involved in colonization (Pic), have been shown to inactivate host immune system components.^67^ ^,^ ^72^ EspP is a class-1 serine protease AT described in EPEC and related enteric *E. coli* backgrounds.^58^ In STEC, a related EspP homolog has been described as PssA (protease secreted by STEC).^68^ Like other SPATEs, EspP undergoes proteolytic processing during secretion, releasing its passenger domain to act extracellularly. One of the key functions of the EspP passenger domain is the cleavage of host substrates, including pepsin A, and human coagulation factor V.^68^ Cleavage of factor V can impair coagulation and has been linked to the pathogenic effects of EspP in enteric infection. Moreover, EspP cleaves the complement system components: C3, C3b, and C5, interfering with the complement cascade and disrupting the first line of host immune defenses.^67^ At the same time, EspP does not target complement regulatory proteins like Factor H (FH) and Factor I (FI), preserving their functionality, which allows intact FH and FI to assist bacteria in evading complement activation via the alternative pathway.^67^ Beyond its effects on host substrates, EspP contributes to bacterial adherence and biofilm establishment by promoting the formation of rope-like adhesive aggregates, which may enhance colonization and potentially support persistence.^69^ Studies show that EspP also exhibits proteolytic activity against T3SS translocator components.^70^ Downregulation of T3SS in this mechanism leads to further promotion of adherence to host tissues.^70^

Pic is a class-2 SPATE described in EAEC and, as PicU, in selected UPEC strains.^71^ ^,^ ^120^ Following secretion and passenger-domain release, Pic contributes to serum resistance and protection from complement-mediated bacteriolysis by degrading key complement proteins involved in all three activation pathways (classical, lectin, and alternative), including C2, C3, C3b, C4, and C4b (Figure 3).^72^ In contrast, Pic does not cleave C1q, C5, or factor H, demonstrating a degree of substrate specificity.^72^ Pic-mediated cleavage of C3 generates a C3b-like fragment that is further degraded by host regulators (factor I and factor H), reducing complement activation synergistically with host complement molecules.^72^ These activities may favor mucosal colonization in specific pathotype- and model-dependent contexts, but a direct contribution to chronic or recurrent infection has not been established. In addition to its complement-targeting activities, Pic exhibits mucinase activity facilitating the degradation of mucins such as MUC2 and MUC5AC, allowing *E. coli* to penetrate the mucus layer.^57^ Surprisingly, a study of Flores-Sanchez et al. revealed that Pic stimulates rapid mucin secretion in goblet-like cells by intracellular Ca-signaling pathway activation mediated by PLC signal transduction pathway activation, DAG production and IP3 release.^59^ Therefore, this dual activity of Pic, functioning both as a mucus secretagogue and a mucinase, facilitates the generation and penetration of the mucus layer niche, enabling EAEC to reside within the mucus layer and simultaneously access epithelial cells.^59^ Pic also specifically targets O-linked glycan-rich glycoproteins on leukocyte surfaces, disrupting proteins involved in adhesion, migration, and inflammatory responses.^79^ A key example is CD43 expressed across nearly all hematopoietic cell lineages, including those in the bone marrow, thymus, and peripheral lymphoid tissues. In its unprocessed form, CD43 functions to prevent leukocyte adhesion to neighboring cells, thereby enabling effective migration in response to chemokine signaling. However, cleavage of CD43 alters its normal regulatory function, thereby promoting leukocyte adhesion to adjacent cells.^79^ This highlights the complexity and multitargeted character of pathogenic strategy facilitating Pic functionality, potentially paralyzing local leukocyte-mediated functions: trafficking, migration, and chemoattractant responding.

**Figure 3.**
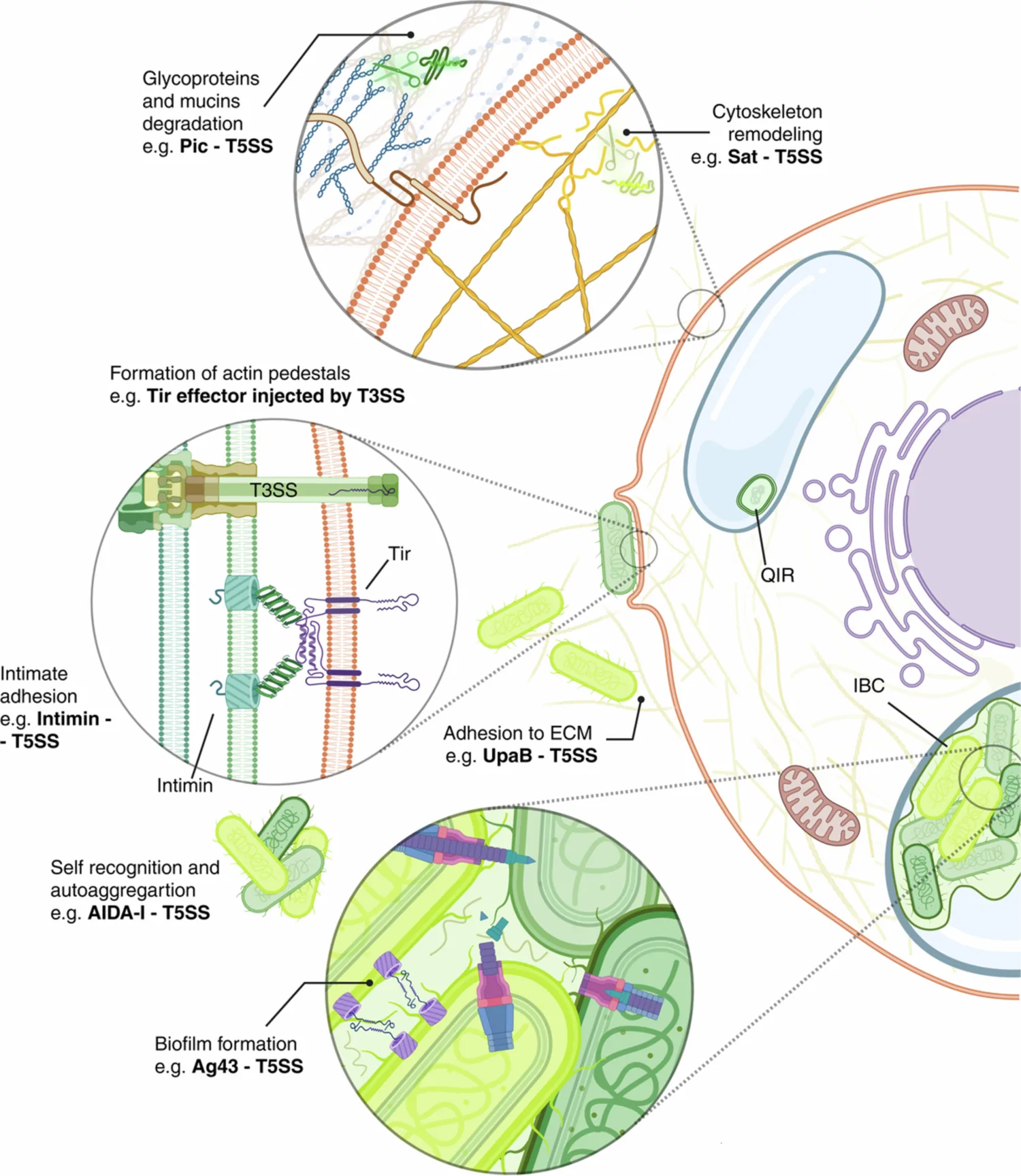
Selected *E. coli* secretion-system-associated processes relevant to host colonization and niche establishment. The illustrated processes have been demonstrated in different pathotypes, strain backgrounds, and experimental models. QIR (Quiescent Intracellular Reservoir) and IBC (Intracellular Bacterial Community) are shown as UPEC-specific reservoir contexts.

Another example of a class-2 SPATE is the non-cytotoxic hemoglobin protease, also known as hemoglobin-binding protein (Hbp), which is expressed in ExPEC and APEC strains and contributes to evasion of host immune responses.^80^ Hbp is closely related to temperature-sensitive hemagglutinin (Tsh); the two homologs differ by only two amino acids and exhibit nearly identical functions. Following Otto et al.^80^ we use the designation Hbp throughout this review.

Similarly to Pic AT, Hbp exhibits proteolytic properties towards mucin, coagulation factor V, and O-glycosylated proteins (CD43, CD45, CD93, CD162, and CXCL1), thereby modifying leukocyte physiology and functionality (Figure 2).^80^ ^,^ ^81^ Furthermore, the most distinctive function of Hbp relates to survival and the acquisition of nutrients critical for growth during infection in a challenging environment. Hbp facilitates iron acquisition for bacterial cells through heme release from hemoglobin.^81^ Iron availability in host cells is tightly regulated and this element is often sequestered to limit bacterial proliferation. By acquiring iron through Hbp, *E. coli* can sustain its survival and growth even in iron-limited environments like the urinary tract or during infection-associated inflammation. This correlates with literature reports of the isolation of Hbp protein from clinical strains of UPEC,^82^ but it remains unclear whether this form of iron acquisition contributes directly to persistent or recurrent infection in vivo.

An example of ATs interacting with host immune responses is the Eib family of immunoglobulin-binding proteins, which belong to the group Vc - trimeric autotransporter adhesins (TAA).^121^ Besides playing a role in adherence to epithelial cells, autoagglutination and biofilm formation, these proteins bind the Fc portion of IgG and IgA in a non-immune manner, effectively interfering with normal antibody functions such as opsonization and activation of the complement cascade (Figure 2).^121^ All Eib family members including EibA, EibC, EibD, EibE, EibF, and the newly identified EibG share several features, such as the ability to impart resistance to human serum complement and a tendency to exist as stable multimers.^122^ ^,^ ^123^ By masking the bacterial surface with bound immunoglobulins, Eib proteins may prevent immune recognition and phagocytosis, thereby contributing to recurrence or persistence of infection, but further studies are necessary to prove this conclusion.^124^

*T6SS*

While research on T6SS primarily focuses on its role in interbacterial competition, there is at present no direct evidence that the T6SS enables the bacterium to evade phagocytosis. Rather than mediating immune evasion in the strict sense, the available studies point instead to roles in immune modulation and intracellular survival.^15^ ^,^ ^95^

A study provides insights into the T6SS's role in intracellular survival.^95^ This study demonstrates that EHEC secretes a catalase enzyme, KatN, via its T6SS. This catalase neutralizes reactive oxygen species (ROS) produced by macrophages, which are essential for killing phagocytosed bacteria (Figure 2). By reducing ROS levels, EHEC can survive within macrophages and potentially evade phagocytic killing. This finding supports a role for T6SS-mediated KatN secretion in intramacrophage survival under oxidative stress.^95^

Further research is needed to determine whether T6SS-associated effects on immune-cell function or intracellular survival extend beyond specific experimental models. At present, available evidence supports a role for selected T6SS effectors in immune modulation or oxidative-stress survival. Investigating the potential role of T6SS effectors in phagocytosis evasion, chronic infection, or recurrence will be crucial for clarifying how T6SS contributes to immune evasion in *E. coli.*

(2) Mucosal niche establishment and reservoir formation

*Adherence to host tissues*

*E. coli* T3SS effectors, besides blocking phagocytosis, manipulate cytoskeletal components to form intracellular pedestals, which promote bacterial attachment and colonization.^36^ ^,^ ^89^ This process, mediated by the translocated effector Tir (translocated intimin receptor), binds the bacterial surface adhesin intimin, triggering a signaling cascade that recruits host actin-nucleating factors (e.g., Nck, *N*-WASP, and the Arp2/3 complex) (Figure 3).^88^ ^,^ ^89^ The resulting pedestal anchors the bacterium to the host cell surface while maintaining its extracellular localization, thereby avoiding internalization and lysosomal destruction.^36^ ^,^ ^89^ Interestingly, EPEC and EHEC utilize distinct mechanisms for pedestal formation, with EPEC relying on Tir tyrosine phosphorylation and EHEC employing the effector protein EspF_U_ to bypass this step.^125^ This dual strategy—blocking phagocytic uptake while securing extracellular adhesion—promotes colonization of the intestinal epithelium and could contribute to persistence under some conditions, but has not been directly linked to chronic or recurrent infection.^19^ ^,^ ^116^ In addition, Esp proteins can act as adhesins, mediating the binding to epithelial cells.^16^ ^,^ ^126^ For example, the EspA, EscF, and the translocon pore proteins EspB and EspD are thought to help the bacteria overcome the thick glycocalyx layer that covers the intestinal epithelium. These components work together to form a channel that connects the bacterial cytosol with the host cell membrane, allowing for the translocation of effector proteins.^17^ ^,^ ^18^ ^,^ ^42^ Studies have shown that *E. coli* strains with a nonfunctional T3SS are unable to adhere to enterocytes and are avirulent in infection models. This highlights the importance of T3SS in *E. coli* pathogenesis and its role in establishing infection.^126^ ^,^ ^127^

In addition, T5SS can also mediate host tissue attachment in diverse *E. coli* pathotypes.^55^ Classical ATs like AIDA-I, TibA and Ag43 promote diffuse adherence to epithelial cells and extracellular matrix components, thereby stabilizing intimate attachment of EHEC, EPEC and UPEC to mucosal surfaces.^60,63–65,128^ Serine protease autotransporters like Sat enable immune evasion by cleaving complement proteins, protecting bacteria from bloodstream clearance.^129^ In different *E. coli* pathotypes, T3SS-driven pedestal formation and T5SS-mediated adhesins can each support extracellular colonization of the intestinal or urinary tract epithelium. Any functional cooperation between these systems during persistent or recurrent infection remains to be demonstrated.^130–132^ The role of T6SS in *E. coli* adherence to host tissues is not well established. However, some studies suggest that it may contribute to this process by modulating bacterial interactions with host cells and the surrounding environment.^103^ ^,^ ^104^ ^,^ ^133^ For example, T6SS effectors can modify host cell adhesion molecules or promote bacterial internalization.^103^ ^,^ ^133^ In EHEC, the T6SS has been shown to be involved in intracellular survival and virulence in mice.^95^ This suggests that the T6SS may contribute to EHEC colonization and survival in specific host niches and underscores its importance for competition with other bacteria in the gut microbiome, whereas any direct role in persistence or recurrence remains speculative.^105^ ^,^ ^134^ ^,^ ^135^

*Biofilm-associated persistence and intracellular reservoirs*

Biofilms are complex communities of bacteria encased in an extracellular matrix that provides protection against environmental stresses and host defenses.^54^ ^,^ ^136^ Numerous secretion systems have been implicated in biofilm formation and maturation or at least play an adjuvant role in the structural and physiological development of biofilms (Tables 1–3). However, despite this vast knowledge, many specific aspects of the role of secretion systems in persistent and recurrent infections, particularly in the context of intracellular bacterial communities (IBC) established by diverse *E. coli* pathotypes other than UPEC, remain insufficiently explored.

Although the T3SS is classically associated with acute host-cell manipulation in enteric *E. coli* pathotypes, persistence in *E. coli* infections is more clearly linked to biofilm formation, intracellular bacterial communities, quiescent intracellular reservoirs, and persister-cell development. This is best established in UPEC, where bacteria can form intracellular bacterial communities within bladder epithelial cells and later establish quiescent intracellular reservoirs (QIRs) that survive antibiotic exposure and immune clearance, thereby contributing to recurrent infection.^7^ ^,^ ^137^ ^,^ ^138^ These intracellular biofilm-like communities are supported by adhesion, aggregation, stress tolerance, and regulatory pathways that promote a sessile or low-metabolic state. In this context, c-di-GMP signaling is particularly relevant because elevated c-di-GMP levels promote biofilm-associated physiology, extracellular matrix production, and persister formation, which may link surface attachment and intracellular persistence to antibiotic tolerance and relapse.^139^ Rather than assigning persistence to a single secretion system, current evidence suggests that secretion-system-associated adhesins, toxins, and competition factors can influence biofilm-associated survival, intracellular maintenance, or reactivation in defined models. These effects depend on broader regulatory and stress-response pathways, including c-di-GMP signaling, metabolic adaptation, and host-niche conditions. Although intracellular bacterial communities and quiescent intracellular reservoirs are well described in UPEC bladder infection models, whether similar reservoir-based strategies operate in other *E. coli* pathotypes remains less well established.^140^

Several T5SS proteins contribute to adhesion, autoaggregation, and biofilm-associated phenotypes in *E. coli*, including AIDA-like adhesins, self-associating autotransporters, and trimeric autotransporter adhesins.^141^ These proteins can promote bacterial–bacterial interactions, epithelial attachment, and extracellular matrix binding, thereby supporting surface-associated colonization and biofilm formation. However, biofilm formation should be interpreted as a persistence-associated phenotype only when linked to long-term colonization, antibiotic survival, reservoir maintenance, or relapse in an appropriate model. The most well-characterized autotransporters contributing to biofilm establishment are Self-Associating Auto Transporters (SAATs): Antigen 43 (Ag43), AIDA-I, and TibA (Figure 3). Extensive research has been conducted on their contribution to biofilm maturation, intracellular survival, and host colonization which has been comprehensively described in the literature over the past two decades.^141^ Ag43, encoded by the *flu* gene, is one of the best-characterized autotransporters involved in autoaggregation and biofilm development. This self-associating adhesin enhances bacterial clustering and biofilm maturation, reinforcing the structural integrity of biofilms in both extracellular and intracellular environments.^54^ ^,^ ^60^ ^,^ ^142^ Notably, Ag43 expression has been associated with UPEC intracellular bacterial communities in bladder epithelial cells, suggesting a role in aggregation or community structure within this niche; its direct contribution to recurrence should be interpreted in the context of UPEC-specific reservoir models.^138^ In addition, Ag43-mediated cell aggregation enhances resistance to oxidative stress, shielding *E. coli* from host immune mechanisms such as neutrophil-derived hydrogen peroxide.^143^

AIDA-I and TibA are SAATs that also contribute to *E. coli* biofilm formation, but are rather secreted by diarrheagenic and enterotoxigenic strains.^61^ ^,^ ^62^ These glycosylated adhesins mediate bacterial aggregation and adhesion to host epithelial cells, supporting colonization-associated phenotypes; direct links to long-term persistence or recurrence remain less well defined. Their ability to modulate interspecies interactions suggests an important role in polymicrobial infections, further complicating treatment strategies. Several studies have indicated that SAATs are induced specifically during biofilm growth, and their expression enhances *E. coli* biofilm formation.^143-145^ Studies on clinically isolated strains of UPEC have demonstrated that *E. coli* isolates from recurrent UTIs exhibit enhanced biofilm-forming ability compared to isolates from acute infections, further supporting the role of biofilms in infection relapse.^146^

Among the autotransporters engaged in adhesion, the Upa family of autotransporters functions primarily as adhesins, mediating bacterial attachment to host extracellular matrix (ECM) components.^56^ ^,^ ^66^ ^,^ ^86^ This role appears complementary to SAATs, which primarily mediate cell-to-cell adhesion and self-recognition ^147^ rather than being involved in non-specific initial adhesion. Type V autotransporters such as UpaB and UpaH facilitate adhesion to fibronectin and laminin and may support bladder colonization in UPEC models (Figure 3).^66^ In murine UTI models, deletion of the *upaB* gene significantly reduces early colonization.^56^ Trimeric adhesin (T5cSS) UpaG further contributes to biofilm stability and interbacterial aggregation, enhancing resistance to hydrodynamic forces within the bladder.^86^ ^,^ ^87^ While their direct involvement in intracellular biofilms remains under investigation, their role in promoting strong bacterial attachment to ECM may influence UPEC colonization and biofilm-associated phenotypes, but direct links to IBC maturation or recurrent infection require model-specific evidence.

In parallel to IBCs, QIRs represent a distinct survival strategy in which *E. coli* enters a quiescent state within deeper layers of bladder epithelial cells. Unlike actively growing IBCs, QIRs consist of non-replicating bacteria enclosed within membrane-bound vacuoles.^7^ Host cell differentiation and actin cytoskeletal rearrangements appear to regulate the transition of QIRs into active infection states, triggering bacterial release and subsequent reinfection.^7^ ^,^ ^60^ ^,^ ^148^ This mechanism may contribute to antibiotic treatment failure because bacteria within QIRs can be protected from antibiotic exposure and immune clearance and may later resume growth under permissive conditions. These intracellular reservoir states should be distinguished from phenotypic persister cells, which are defined by antibiotic tolerance without genetic resistance.^7^ ^,^ ^137^

Among T5SS-associated proteins, the vacuolating autotransporter toxins Vat and Sat can modify epithelial-cell physiology. These toxins, members of the SPATEs, induce vacuolation and cytoskeletal remodeling in host epithelial cells, which can promote intracellular survival or dissemination in experimental models.^75^ ^,^ ^78^ These activities may create host-cell conditions that favor bacterial survival or dissemination, but direct roles in QIR formation or recurrent infection require cautious interpretation. Vat, expressed by UPEC, disrupts the urothelial monolayer and increases epithelial permeability (Figure 2). By altering the actin cytoskeleton and tight junctions, it facilitates bacterial invasion and intracellular survival.^76^ ^,^ ^78^ Similarly, Sat exhibits vacuolating activity in bladder and kidney epithelial cells, leading to epithelial damage and bacterial dissemination (Figure 3).^75^ ^,^ ^76^ The ability of these toxins to create intracellular niches further complicates bacterial clearance, suggesting their potential contribution to prolonged or relapsing infections. However, further research into the precise molecular interactions governing these processes is essential to fully understand their contribution and to develop novel treatment strategies aimed at preventing reinfection.

The T6SS has been implicated in biofilm formation in several bacterial species, including *E. coli*.^113^ Evidence from other Gram-negative bacteria illustrates how T6SS activity can influence biofilm-associated competition. In *Vibrio cholerae*, T6SS-mediated killing within biofilms is associated with localized extracellular DNA release, whereas the response regulator VxrB coordinates T6SS gene expression and biofilm matrix production.^149^ These findings illustrate a possible link between T6SS-mediated competition and biofilm architecture, but their relevance to *E. coli* biofilm-associated persistence remains to be demonstrated. Another example is that T6SS-mediated killing of neighboring bacteria can alter the structure and composition of the biofilm. This can be beneficial for the bacteria possessing the T6SS, allowing them to dominate the biofilm and access more resources.^150^

In addition, T6SS has an important role in intermicrobial interactions. Comparative studies in other Gram-negative pathogens, including *Vibrio cholerae* and *Salmonella enterica*, show that T6SS-mediated antibacterial activity can shape intestinal colonization by targeting competing members of the gut microbiota.^134^ ^,^ ^135^ In *E. coli*, available evidence suggests that T6SS may similarly contribute to strain-dependent interbacterial competition, niche fitness, and persistence-associated interactions within polymicrobial environments, although the ecological relevance of these activities remains less well defined.

*Persistence in the host*

Persister cells represent phenotypically altered bacterial subpopulations that can survive otherwise bactericidal antibiotic exposure without acquiring genetic resistance.^151^ This bet-hedging survival strategy allows a small fraction of cells to enter a transient growth-arrested or low-activity state, tolerate antibiotic stress, and resume growth once antibiotic pressure is removed.^152^ In *E. coli*, persister-cell biology should be distinguished from biofilm-associated protection, intracellular reservoirs, poor antibiotic penetration, and clinical recurrence. These processes may overlap in some infection settings, but they are not equivalent.

Among secretion-system-linked mechanisms, the clearest experimental connection is contact-dependent inhibition (CDI). Ghosh et al. demonstrated that CDI systems in the commensal gut isolate EC93 and the UPEC strain 536 contribute to antibiotic-tolerant persister-cell formation through direct cell-cell interactions.^85^ CDI-mediated growth inhibition can induce a persister-like state in susceptible cells within dense clonal populations, and this effect depends on the balance between toxin delivery and cognate immunity.^85^ Notably, CDI-mediated persister formation is population density-dependent, with higher persister fractions arising in dense, clonal communities.^85^ These findings suggest that CDI can link interbacterial interaction to antibiotic-tolerant survival under defined high-density conditions, although broader roles in intracellular reservoirs, treatment failure, or recurrent infection remain indirect and require model-specific validation.

In vitro studies of antibiotic-induced persisters have traditionally emphasized dormancy, but this assumption may not apply to all intracellular antibiotic-tolerant populations.^151^ This assumption is not necessarily absolute in case of intracellular persisters, as shown by Staples and colleagues on the example of non-growing, antibiotic-tolerant *Salmonella enterica* subsp. *enterica* serovar Typhimurium persister cells which actively subverted macrophage host cells.^153^ During macrophage infection, *S. enterica* antibiotic persisters retain transcriptional, translational, and metabolic activity and, moreover, translocate pathogenicity island 2 (SPI-2) type III secretion system effectors into the eukaryotic cytosol to reprogram the host cell into a non-inflammatory and infection-permissive state.^153^ Although similar behavior has not been demonstrated for *E. coli*, this finding raises the possibility that secretion-system activity may also contribute to the host-modulating capacity of intracellular *E. coli* persisters. This remains speculative and should be tested directly in *E. coli* infection models.

A related, but distinct, question concerns factors that support intracellular survival of *E. coli* in phagocytic cells. OmpA-positive *E. coli* can bind, enter, and survive within murine and human macrophages and monocytes, suppress selected chemokine or cytokine responses, and inhibit macrophage apoptosis through induction of the anti-apoptotic factor Bcl-xL.^154^ ^,^ ^155^ OmpA has also been reported to influence secretion of T3SS proteins such as EspA and EspB in EHEC without reducing expression of LEE-encoded T3SS genes.^156^ These findings suggest a possible link between envelope-associated factors, secretion activity, and intracellular host-cell modulation, but they should be interpreted as an open research question rather than evidence that *E. coli* persisters actively reprogram host cells during antibiotic exposure.

(3) Potential functional interplay and open questions on secretion-system co‑regulation

The interactions discussed in this subsection are presented as hypothesis-generating observations rather than as evidence of a coordinated secretion-system program. Each has been documented individually, but their integration into a single regulatory network has not been demonstrated. Evidence from separate studies suggests possible functional convergence between secretion-system-associated processes or parallel responses to shared host cues. Whether these observations reflect direct cross-regulation, coordinated expression, or independent context-dependent responses remains unknown. Nevertheless, comparative analysis of distinct secretion systems suggests that their activity may be context-dependent and modulated by host or environmental cues, potentially allowing *E. coli* to adjust its interaction strategies across different colonization and infection settings.

Particularly interesting is the “pseudo-antagonistic” interaction between two major virulence systems, the T5SS and T3SS, mentioned above. The proteolytic activity of the extracellular serine protease EspP against T3SS translocon components constitutes a form of negative regulation that can dampen T3SS‑dependent host-cell interactions.^70^ However, this effect may be offset by EspP’s additional functions, including rope‑like aggregate formation and complement cleavage, which facilitate adherence, biofilm formation, and immune evasion.^67^ ^,^ ^69^ In this way, EspP activity could shift the balance from strongly invasive, T3SS‑dominated interactions toward more colonization‑oriented strategies in specific niches, although any contribution to chronic or recurrent infection in *E. coli* remains to be demonstrated. Conversely, EspP appears to exhibit functional synergy with the autotransporter Pic in the degradation of key complement cascade components, especially C3 and C3b, thereby protecting bacteria from complement-mediated lysis.^67^ EspP activity preserves the integrity of the complement regulators Factor I and Factor H, which are essential for the degradation of Pic-derived C3b-like fragments and for co-opting host regulatory molecules to suppress complement activation and enhance inhibition of the alternative pathway.^72^ ^,^ ^157^ Thus, both autotransporters enhance immune evasion not only through direct proteolysis, but also via synergistic collaboration with host immune regulators.

Beyond transcriptional control, one possible model is that T3SS and T6SS activities could, in principle, act in a complementary fashion to promote intracellular survival. As described above, the T6SS enhances survival within macrophages by secreting the catalase KatN, which neutralizes reactive oxygen species (ROS) generated during the oxidative burst in specific EHEC infection models.^95^ By analogy, the T3SS activity has been implicated in maintaining persister-like intracellular cells in *Salmonella* ^158^ and could, in principle, modulate host trafficking or inflammatory signaling in ways that prevent bactericidal responses in *E. coli.* Together, these systems might collectively resemble an intracellular protection module in some infection settings. Although few studies have explicitly linked these mechanisms, their coexistence across multiple infection models is consistent with, but does not establish, such a coordinated strategy, which remains to be tested directly. At present, however, these mechanisms have been documented separately, and there is no direct evidence that they operate as a coordinated program in *E. coli.*

Overall, the cited studies suggest that secretion-system activities can be context-dependent and may respond to overlapping host or microbial cues. However, evidence that T3SS, T5SS, and T6SS operate as a coordinated persistence program in *E. coli* remains lacking. Future studies should directly test whether these systems are co-regulated, co-expressed, or functionally linked during long-term colonization, antibiotic exposure, or recurrence.

## IV. From gut colonization to recurrence: evidence limits and future directions

*E. coli* secretion systems contribute to several biological processes that can influence long-term colonization, mucosal niche formation, microbiota competition, antibiotic survival, and recurrent infection. However, the evidence does not currently support a single unified model in which T3SS, T5SS, and T6SS act as a coordinated secretion-system network driving persistence across all *E. coli* pathotypes. Their roles are better understood as pathotype-specific, niche-specific, and evidence-dependent.

T3SS is best established as an epithelial interaction system in enteric pathotypes such as EPEC and EHEC, where it promotes intimate attachment, cytoskeletal remodeling, barrier disruption, and modulation of host signaling. These activities may facilitate colonization and create a mucosal interface favorable to bacterial persistence, but they should not be interpreted as direct evidence for chronic infection or recurrence without appropriate model-specific support. T5SS provides one of the clearest links to mucosal persistence-associated phenotypes because many autotransporters and related proteins promote adhesion, aggregation, biofilm formation, mucin interaction, complement resistance, and epithelial damage. These functions are particularly relevant to reservoir formation and tissue colonization, especially in ExPEC and UPEC contexts, but they also overlap with general colonization and virulence mechanisms. T6SS is most strongly supported as an ecological and microbiota-facing system. Its antibacterial effectors, immunity proteins, and contact-dependent killing activity can influence interbacterial competition and niche occupation within polymicrobial environments. Links between T6SS and recurrence or chronic infection in *E. coli* remain more indirect and should be tested in strain- and niche-defined models.

A major challenge for the field is to separate persistence-associated mechanisms from general virulence traits. Adhesion, immune modulation, epithelial injury, biofilm formation, and interbacterial competition can all support colonization, but they do not automatically demonstrate persistence, persister-cell formation, or clinical relapse. Future studies should therefore define the biological endpoint carefully: persistent colonization, persistent infection, phenotypic antibiotic tolerance, biofilm-associated survival, intracellular reservoir formation, relapse, or reinfection. This distinction is especially important when comparing enteric *E. coli* pathotypes with UPEC and other ExPEC lineages, because mechanisms established in bladder reservoir models cannot be directly transferred to intestinal carriage or systemic infection.

Several key evidence gaps remain. It is still unclear whether T3SS, T5SS, or T6SS genes are consistently enriched in recurrent clinical isolates, whether these systems are expressed during long-term colonization, whether secretion activity persists after antibiotic exposure, and whether secretion-system mutants show reproducible defects in relapse or reservoir reseeding *in vivo*. It is also unclear how secretion systems interact with better-established persistence determinants, including type 1 fimbriae, curli, capsule, LPS modification, iron acquisition systems, toxins, stringent and general stress responses, c-di-GMP signaling, biofilm matrix production, and metabolic adaptation. Addressing these questions will require longitudinal clinical isolate collections, pathotype-resolved comparative genomics, *in vivo* expression analyzes, polymicrobial gut models, and infection systems that distinguish relapse from reinfection.

Overall, T3SS, T5SS, and T6SS should be viewed as context-dependent interaction modules rather than universal drivers of *E. coli* persistence. Their importance lies in the fact that they connect bacterial physiology to host epithelial responses, immune pressures, microbial competition, and reservoir stability. A critical, evidence-calibrated understanding of these systems may help identify when secretion-system activity is merely associated with colonization and when it causally contributes to persistent or recurrent infection. This distinction will be essential for developing future anti-virulence or reservoir-disrupting strategies that complement, rather than replace, conventional antimicrobial approaches.

## Data Availability

No data were generated in this study.
